# Coronavirus disease 2019 induces multi‐lineage, morphologic changes in peripheral blood cells

**DOI:** 10.1002/jha2.44

**Published:** 2020-06-29

**Authors:** Florian Lüke, Evelyn Orsó, Jana Kirsten, Hendrik Poeck, Matthias Grube, Daniel Wolff, Ralph Burkhardt, Dirk Lunz, Matthias Lubnow, Barbara Schmidt, Florian Hitzenbichler, Frank Hanses, Bernd Salzberger, Matthias Evert, Wolfgang Herr, Christoph Brochhausen, Tobias Pukrop, Albrecht Reichle, Daniel Heudobler

**Affiliations:** ^1^ Department of Internal Medicine III, Haematology and Oncology University Hospital Regensburg Regensburg Germany; ^2^ Institute for Clinical Chemistry and Laboratory Medicine University Hospital Regensburg Regensburg Germany; ^3^ Department of Anesthesiology University Hospital Regensburg Regensburg Germany; ^4^ Department of Internal Medicine II University Hospital Regensburg Regensburg Germany; ^5^ Institute of Clinical Microbiology and Hygiene University Hospital Regensburg Germany; ^6^ Department of Infection Prevention and Infectious Diseases University Hospital Regensburg Regensburg Germany; ^7^ Institute of Pathology University of Regensburg Regensburg Germany

**Keywords:** blood differential count, COVID‐19, hemato‐morphology, peripheral blood smear, SARS‐CoV‐2

## Abstract

The clinical course of coronavirus disease 2019 (COVID‐19) varies from mild symptoms to acute respiratory distress syndrome, hyperinflammation, and coagulation disorder. The hematopoietic system plays a critical role in the observed hyperinflammation, particularly in severely ill patients. We conducted a prospective diagnostic study performing a blood differential analyzing morphologic changes in peripheral blood of COVID‐19 patients. COVID‐19 associated morphologic changes were defined in a training cohort and subsequently validated in a second cohort (n = 45). Morphologic aberrations were further analyzed by electron microscopy (EM) and flow cytometry of lymphocytes was performed. We included 45 COVID‐19 patients in our study (median age 58 years; 82% on intensive care unit). The blood differential showed a specific pattern of pronounced multi‐lineage aberrations in lymphocytes (80%) and monocytes (91%) of patients. Overall, 84%, 98%, and 98% exhibited aberrations in granulopoiesis, erythropoiesis, and thrombopoiesis, respectively. Electron microscopy revealed the ultrastructural equivalents of the observed changes and confirmed the multi‐lineage aberrations already seen by light microscopy. The morphologic pattern caused by COVID‐19 is characteristic and underlines the serious perturbation of the hematopoietic system. We defined a hematologic COVID‐19 pattern to facilitate further independent diagnostic analysis and to investigate the impact on the hematologic system during the clinical course of COVID‐19 patients.

## 1 INTRODUCTION

The coronavirus disease 2019 (COVID‐19) caused by severe acute respiratory syndrome coronavirus 2 (SARS‐CoV‐2) has emerged as a global challenge in respect of medical therapy as well as widespread socioeconomic consequences [1, 2]. With no vaccine or highly effective treatment available to date, the major strategies to combat the disease consist of mitigating transmission by containment efforts as well as ameliorating morbidity and mortality in severely ill patients by drugs limiting viral replication or modulating dysbalanced immune response [3, 4]. In terms of smart containment strategies widespread testing with high sensitivity and specificity is a prerequisite. However, the “gold standard” test by real‐time polymerase chain reaction (RT‐PCR) takes several hours in time and requires substantial human und laboratory resources. Moreover, the percentage of false negative pharyngeal samples is quite high partly due to the focused selection of a single compartment for routine diagnostics [5]. Additional laboratory findings supporting diagnosis and enabling follow up diagnostics to evaluate the course of the disease of COVID‐19 would be helpful, especially when they are everywhere and rapidly available and cost effective [6].

There is evidence of SARS‐CoV‐2 affecting multiple organs, including the hematopoietic and immune system [7, 8]. In COVID‐19 lymphocytopenia, neutrophilia, eosinopenia, thrombocytopenia, or thrombocytosis are common findings in blood counts [1, 7, 9, 10, 11, 12, 13, 14]. A low lymphocyte/neutrophil ratio at diagnosis may be even prognostic for developing a severe course of disease [8]. Moreover, some studies report sporadic observations about morphologic changes in peripheral blood like “reactive” or plasmocytoid lymphocytes, abnormal monocytes/granulocytes/thrombocytes, and leukoerythroblastic reaction [15, 16, 17, 18].

Yet, no systematic evaluation of morphologic changes in hematopoietic cells in COVID‐19 patients has been performed. Such observed changes in peripheral blood cells could in principal represent a specific SARS‐CoV‐2‐mediated impact on hematopoiesis. This assumption is supported by hematologic findings during other viral diseases (ie, mononucleosis, dengue fever, etc), which help clinicians to secure a correct diagnosis [19, 20].

Our prospective study aimed to provide a comprehensive description of all morphologic aberrations in blood cells associated with COVID‐19. We demonstrate that a systematic search for this hematologic pattern can provide a straightforward and easy‐to‐perform additional diagnostic tool. Moreover, COVID‐19‐induced hematologic changes might provide hints to what extent the hematopoietic system contributes to the pathophysiology of this new viral disease.

## 2 METHODS

### 2.1 Patients

In this prospective study, 45 patients suffering from COVID‐19 treated at the University Hospital Regensburg were included. SARS‐CoV‐2 infection was confirmed by RT‐PCR. The flow cytometric analysis also included eight healthy sex‐ and age‐matched individuals with similar age and sex as controls. We obtained epidemiological, demographic, clinical, laboratory, management, and outcome data from patients' medical records. Clinical outcomes were followed up to May 08, 2020. The study was approved by the Ethics Committee of the University Regensburg (ethics statement No.: 20‐1785‐101) and written informed consent was obtained from all patients and healthy controls. The data that support the findings of this study are available on request from the corresponding author. The data are not publicly available due to privacy or ethical restrictions.

### 2.2 Blood differential

Peripheral blood samples, containing potassium salt of ethylenediaminetetraacetic acid (K_2_‐EDTA) as anticoagulant, were drawn for diagnostic and/or follow‐up purpose. The smears were stained for light microscopy according to Pappenheim (combination of Jenner‐May‐Grünwald and Giemsa). Two laboratory technicians with special training in hematology, as well as two experienced specialists in hematology performed microscopic blood differentials according to standard procedures. Standardized nomenclature and scoring of cellular features were applied according to the recommendations of the International Council for Standardization in Haematology (ICSH) [21]. For the assessment of qualitative aberrations in blood cells a semi‐quantitative scoring system was used, providing 1+ (light), 2+, 3+, and 4+ (highest) grades of alterations. Routine blood differentials were assessed for distinct SARS‐CoV‐2 associated changes defined in the initial training subset. For a clear discrimination of SARS‐CoV‐2 associated changes in hematopoietic cells from those of other etiologies, the term “aberrant” was used exclusively for COVID‐19; in contrast, the term “reactive/atypical lymphocyte” was applied to describe lymphocytes with a benign/malignant etiology.

### 2.3 Electron microscopy

For electron microscopy analyses, leukocytes and mononuclear cells were isolated from selected whole blood samples by density gradient centrifugation on Ficoll‐Paque. The cell pellet was fixed with buffered glutaraldehyde (Karnovsky fixative), and then enclosed in low melting agarose. The embedding process was performed in standardized, automated manner by use of the LYNX microscopy tissue processor (Reichert‐Jung, Wetzlar, Germany). Semi‐thin‐sections and ultra‐thin sections (80 nm) were cut using the Reichert Ultracut S Microtome (Leica‐Reichert, Wetzlar, Germany). Ultra‐thin‐sections were contrasted with aqueous 2% uranyl‐acetate and 2% lead‐citrate solution for 10 min each. Electron‐microscopic analysis was performed using the EFTEM LEO 912AB electron‐microscope (Zeiss, Oberkochen, Germany).

### 2.4 Immunophenotyping of circulating lymphocyte subsets

A detailed description of multiparametric immunophenotyping is given in the Supporting Information.

## 3 RESULTS

### 3.1 Patient characteristics

The patient cohort consisted of 45 hospitalized patients suffering from COVID‐19. Due to longitudinal sampling, altogether 76 samples were analyzed (multiple samples in 15 patients, median three samples, range 2‐6). The median age was 58 years (ranging from 21 to 77 years) and most patients were male (Table 1). Notably, the cohort consisted of severely ill patients. Thirty‐seven patients were treated in an intensive care unit (ICU) of whom 36 needed mechanical ventilation, 11 even needed extracorporeal membrane oxygenation (ECMO). No antiviral agents were used. Four patients received anakinra due to severe secondary hemophagocytic lymphohistiocytosis (HLH). In peripheral blood counts, 41% of the samples showed leukocytosis, 83% lymphocytopenia, 88% monocytopenia, 17% thrombocytopenia, and 37% thrombocytosis. Of note, three patients suffered from chronic lymphocytic leukemia as concomitant disease. At the time of sample collection, median duration of disease was 24 days ranging from 6 to 54 days (Figure S1).

**TABLE 1:** Data of patient demographics, basic laboratory findings and list of comorbidities

Demographic	No. of patients (%)
Patients	45
Samples	76
Age, years	58 (21‐77)
*Sex*	
Male	30 (67)
Female	15 (33)
ICU care	37 (82.0)
On respirator	36 (80)
ECMO	11(24.4)

Laboratory findings	Median (min; max)
Leucocytes (nL^−1^)	8.73 (1.69; 116.15)*
Lymphocytes (nL^−1^)	1.25 (0.25; 76.66)
Monocytes (nL^−1^)	0.65 (0.0; 2.74)
Hemoglobin (g/dL)	8.6 (6.9; 13.1)
Platelets (nL^−1^)	278 (37; 770)
LDH (U/L)	369 (167; 1080)
C‐reactive protein (mg/L)	64.35 (3.2; 372)
Procalcitonine (ng/mL)	0.47 (0.06; 27.7)

Comorbidities	No. of patients (%)
None	10 (22.2)
Arterial Hypertension	14 (31.1)
Diabetes mellitus	9 (20.0)
Atrial fibrillation	4 (8.9)
Obstructive sleep apnea	4 (8.9)
Carcinoma in patient history	4 (8.9)
Adipositas	3 (6.7)
Chronic lymphocytic leukemia	3 (6.7)
Liver cirrhosis	3 (6.7)
Hypercholesterinemia	2 (4.4)
Hepatitis C	2 (4.4)
Organ transplant	2 (4.4)
Coronary heart disease	2 (4.4)
Non‐Hodgkin lymphoma	1 (2.2)

Abbreviations: ECMO, extracorporeal membrane oxygenation; LDH, lactate dehydrogenase.

^*^The cohort contained three patients with chronic lymphocytic leukemia. This explains the high maximum counts of leukocytes.

### 3.2 Alterations in the peripheral blood

#### 3.2.1 Blood differential

**FIGURE 1:**
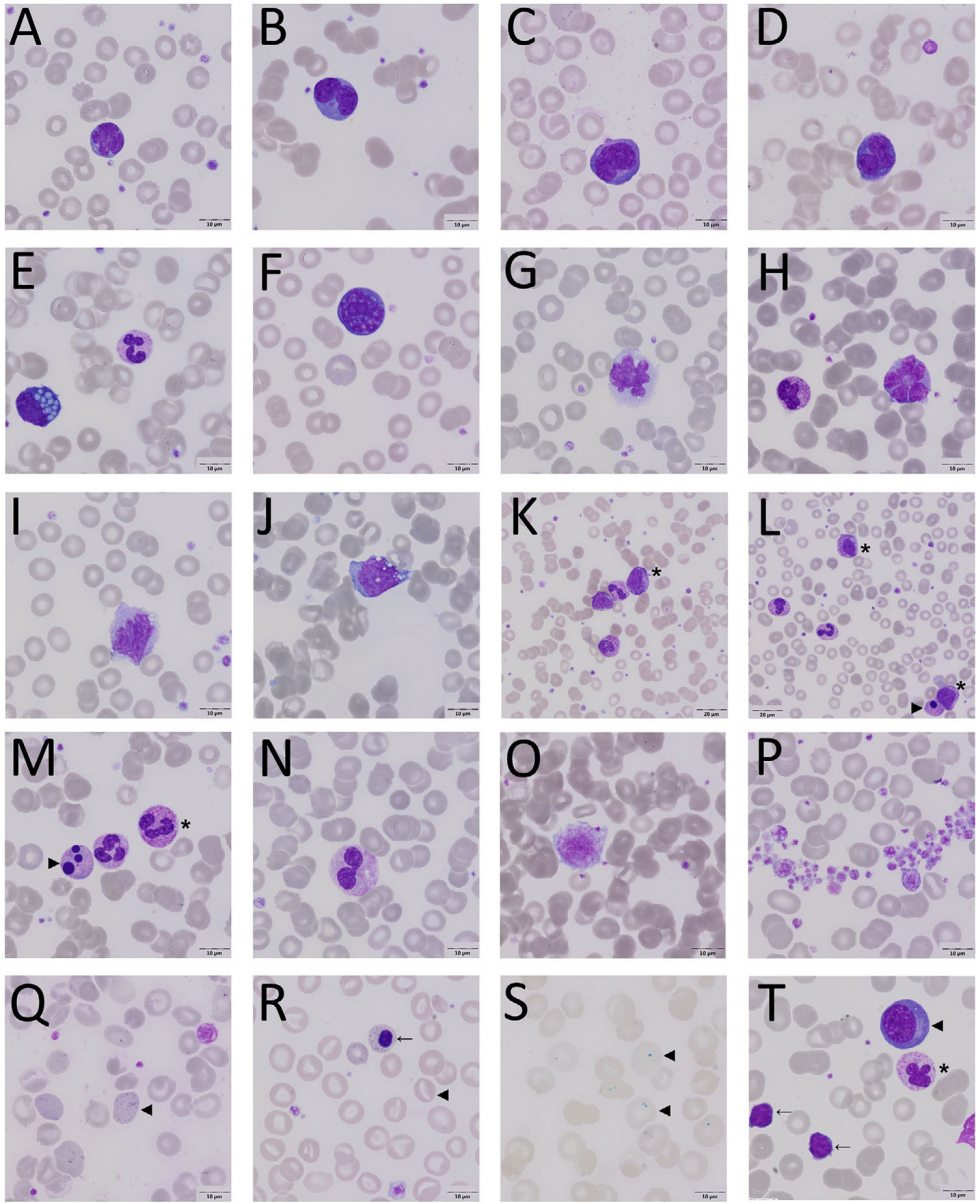
Morphologic aberrations in blood smears in COVID‐19. A and B, Aberrant lymphocytes with multi‐lobulated nuclei and large cytoplasmatic granula (B); C and D, aberrant lymphoplasmocytoid cells; E, Mott cell; F, enlarged lymphocyte with basophilic cytoplasm, undergoing apoptosis with signs of karyolysis; G and H, lympho‐monocytoid cells with aberrant lobulated nuclei; I and J, monocytes with aberrant nuclei (clumped chromatin) and basophilic cytoplasms; K and L, left shift with myelocytes/metamyelocytes (*) and hypergranulation; M, granulocytes with hypergranulation (*); L and M, (◄) apoptotic cells; N, neutrophil granulocyte with hypolobulated nucleus, pseudo‐Pelger‐Huët anomaly; O, giant platelet with cytoplasmic vacuoles; P, giant platelets and aggregated platelets; Q and R, aberrant erythropoiesis: anisocytosis of RBCs, basophilic stippling (Q◄), stomatocytes (R◄), target‐cell and nucleated RBC (←); S, Prussian blue staining showing large iron‐containing deposits (◄); T, patient with CLL: two atypical lymphocytes (←) (CLL) and one large aberrant lymphocyte with basophilic cytoplasm, likely plasmablast with clumped chromatin(◄), neutrophil granulocyte with hypergranulation and aberrant segmentation of the nucleus

The first observations of distinct morphologic changes in blood smears of COVID‐19 patients led to the analysis of an initial (training) cohort (n = 15 COVID‐19 patients). In this initial group of patients, marked morphologic anomalies in all three hematologic lineages were found (Figure 1). In particular, “aberrant lymphocytes” differing from reactive lymphocytes that are commonly seen in other viral diseases such as dengue fever and infectious mononucleosis, as well as “aberrant” monocytes were observed (Figure 1). Moreover, marked aberrations in granulo‐, erythro‐, and thrombopoiesis were also seen (detailed composition of aberrations is shown in Table S1).

In order to further evaluate whether these morphologic changes (Figure 1; Table S1) are associated with COVID‐19, we performed a prospective analysis in a larger cohort of COVID‐19 patients using an “experimental” blood differential in which all suspected COVID‐19 associated aberrations (Table S1) were quantified separately. The results are demonstrated in Table 2. Median differential counts for “aberrant” lymphocytes and “aberrant” monocytes were 1% and 3%, respectively. Pronounced aberrations in all lineages were observed.

**TABLE 2:** Light microscopic blood differential blood smears of COVID‐19 patients

Cell Type	Median (min; max)
Blasts (%)	0 (0; 2)
Promyelocytes (%)	0 (0; 4)
Myelocytes (%)	0.5 (0; 5)
Metamyelocytes (%)	0 (0; 9)
Band neutrophils (%)	2.5 (0; 32)
Segmented neutrophils (%)	72 (5; 89)
Eosinophils (%)	1 (0; 31)
Basophils (%)	0 (0; 2)
Aberrant basophils (%)	0 (0; 5)
Lymphocytes* (%)	11.5 (0; 52)
aberrant lymphocytes* (%)	*1 (0; 23)*
Atypical lymphocytes* (%)	0 (2; 66)
Reactive lymphocytes (%)	0 (0; 2)
Plasma cells (%)	0 (0; 2)
Monocytes (%)	5 (0; 15)
*Aberrant Monocytes* (%)	*3 (0; 8)*
Erythroblasts (per 100 WBC)	0 (0; 22)

Morphologic feature	Median (min; max)
Apoptosis	− (−; ++)
Hypergranulated neutropils	+ (−; +++)
Hypogranulated neutrophils	− (−; +)
Neutrophils with Pelger‐Huët nuclear alteration	− (−; ++)
Döhle Bodies	− (−; +)
Polychromasia of red blood cells	+ (−; ++)
Anisocytosis of red blood cells	+ (−; +++)
Macrocytosis of red blood cells	− (−; ++)
Microcytosis of red blood cells	+ (−; ++)
Stomatocytes	− (−; +++)
Rouleaux formation	− (−; +++)
Aanisocytosis of platelets	+ (−; +++)
Giant platelets	+ (−; ++)

^*^The cohort contained three patients with chronic lymphocytic leukemia. This explains the high maximum counts of lymphocytes and atypical lymphocytes.

In the entire cohort “aberrant” lymphocytes and “aberrant” monocytes were present in 36 patients (80%) and 41 patients (91%), respectively (Table 3). Interestingly, “classical,” reactive/activated lymphocytes were observed only in five patients (9%). In addition, 84%, 98%, and 98% of patients exhibited aberrations in granulopoiesis, erythropoiesis, and thrombopoiesis, respectively. Particularly, anomalies like Pseudo‐Pelger Hüet neutrophils, nucleated red blood cells, and giant platelets were found frequently showing a serious perturbation of the entire hematopoiesis with features usually seen in hematologic neoplasms like myelodysplastic syndrome (MDS) and myeloproliferative neoplasms (MPN). A left shift in myeloid cells was observed in 67% of patients.

**TABLE 3:** Frequency of morphologic changes in blood smears of COVID‐19 patients

	All patients and samples		Still SARS‐CoV‐2 positive patients and samples		After negative SARS‐CoV‐2 test patients and samples	
Morphology	Patients (%); n = 45	Samples (%); n = 76	Patients (%); n = 36	Samples (%); n = 54	Patients (%); n = 14	Samples (%); n = 22
Aberrant lymphocytes	36 (80)	56 (74)	29 (81)	38 (70)	12 (86)	18 (82)
Aberrant monocytes	41 (91)	69 (91)	32 (89)	48 (89)	14 (100)	21 (95)
Left shift	31 (67)	48 (63)	26 (72)	37 (69)	8 (57)	11 (50)
Aberrant granulopoiesis	38 (84)	64 (84)	32 (89)	47 (87)	11 (79)	17 (77)
Hypergranulation	35 (78)	58 (76)	30 (83)	43 (80)	10 (71)	15 (68)
Pseudo‐Pelger Huët neutrophils	21 (47)	30 (39)	19 (53)	25 (46)	4 (29)	5 (23)
Aberrant erythropoiesis	44 (98)	75 (99)	35 (97)	53 (98)	14 (100)	22 (100)
Nucleated red blood cells	9 (20)	16 (21)	8 (22)	11 (20)	3 (21)	5 (23)
Aberrant thrombopoiesis	44 (98)	75 (99)	35 (97)	53 (98)	14 (100)	22 (100)
Giant thrombocytes	39 (87)	67 (88)	30 (83)	46 (85)	14 (100)	21 (95)
Apoptotic cells	17 (38)	22 (29)	14 (39)	14 (26)	6 (43)	8 (36)

In our cohort, 14 patients developed negativity for SARS‐CoV‐2 RNA in respiratory material samples. In order to evaluate whether the elimination of the virus had any impact on the observed changes in the peripheral blood, we performed an additional analysis classifying patients/samples as “still SARS‐CoV‐2 positive” or “after negative SARS‐CoV‐2 test.” Comparing these two subgroups, we observed a significant decrease in the left shift of myeloid cells as well as a decrease in hypergranulation and the presence of Pseudo‐Pelger Huët neutrophils after virus elimination while all the other anomalies showed no change (Table 3; Figure S2). Similarly, we observed a significant decrease in C‐reactive protein (CRP) (Figure S3) representing a reduction in systemic inflammation. Thus, the left shift and “aberrations” in the granulopoiesis might be caused by the SARS‐CoV‐2‐induced systemic inflammatory response.

#### 3.2.2 Electron microscopy

**FIGURE 2:**
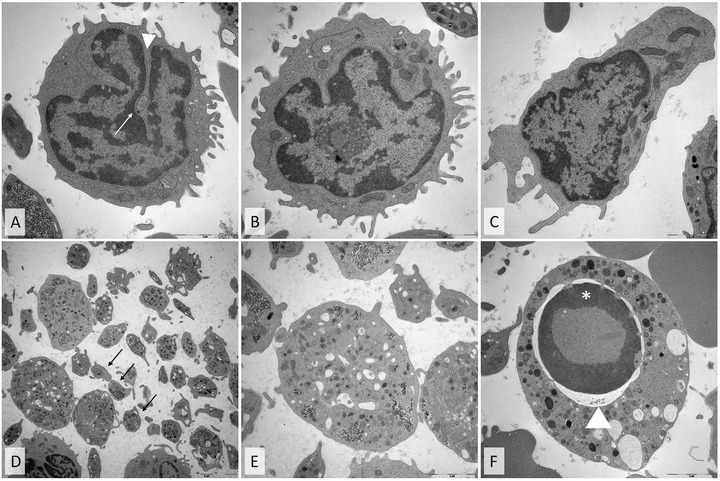
Representative ultrastructural changes of aberrant cells. A, Lymphocyte with invagination of the cytoplasm (arrowhead) containing a mitochondrion (arrow). B, Lymphocyte with highly lobulated nucleus. C, Markedly elongated lymphocyte. D and E, Ultrastructural change of platelets and granulocytes: D, a mixture of normally sized platelets (arrows) and giant platelets (E) was obvious. F, Granulocyte, showing apoptosis with nuclear pycnosis with shrinking (arrowhead) and chromatin condensation (*)

To assess the ultrastructural equivalents of the distinctive aberrations seen by light microscopy, electron microscopy on peripheral blood cells was performed. In transmission electron microscopy, numerous lymphocytes showed markedly nuclear invaginations, sometimes containing cell organelles such as mitochondria (Figure 2). In addition, lymphocytes with highly lobulated nuclei were found. Interestingly, elongated lymphocytes could be demonstrated. Furthermore, we could confirm the finding of giant platelets. Among normal platelets 1.5‐3 μm in diameter, enlarged platelets with a dimension of >5 μm were also present. Finally, we identified granulocytes in early stages of apoptosis, exhibiting hypercondensed chromatin and the beginning of nuclear shrinking.

#### 3.2.3 Quantitative alterations of circulating lymphocyte subpopulations

In addition to the microscopic leukocyte differential, a quantitative analysis of lymphocytes, regarding their major subpopulations, was performed. Relative T cells counts (as % of leucocytes) were significantly lower in the COVID‐19 group than in the healthy control group. There was no difference between the two groups in respect of absolute T cell and B cell counts. Interestingly, there was a significantly lower absolute NK cell count in the COVID‐19 group as compared to the healthy control group (Table S2). In addition to the quantification of circulating lymphocyte subpopulations, cell surface expression of all major antigens (ie, CD3, CD4, CD5, CD8) on T cells and their subsets were determined by flow cytometry. We found significant higher expression of CD3, CD5, and CD8 on the CD8^+^ T cell subpopulation in the COVID‐19 group, as compared to the healthy control group (Figure S4).

## 4 DISCUSSION

Our morphologic observations reveal that COVID‐19 is associated with hematologic multi‐lineage changes of all cellular components of the peripheral blood, that is, lymphocytes, monocytes, granulocytes, erythrocytes, and platelets. This is consistent with previous, sporadic reports describing particular aspects like abnormal lymphocytes, plasmocytoid cells, monocytes, granulocytic, or leukoerythroblastic reactions in COVID‐19 patients [15, 16, 17, 18]. Interestingly, while other virus infections (eg, Epstein‐Barr virus or Dengue fever) are known to induce morphologic changes in lymphocytes (termed “reactive lymphocytes” or “virocytes”), these “classical” reactive lymphocytes were found only infrequently in COVID‐19 patients in our study cohort [19, 20]. Electron microscopic investigations revealed the ultrastructural equivalents of the observed changes like nuclear invaginations or apoptotic cells and confirmed the multi‐lineage aberrations already seen by light microscopy.

The observed multi‐lineage hematologic changes are very helpful to shed light on the complex pathophysiology leading to the severe disease pattern in COVID‐19 as our morphological findings in the peripheral blood could reflect the complex immune dysregulation in COVID‐19 patients with respiratory failure [3]. The aberrations in the myeloid compartment with accelerated and dysplastic granulo‐ and monopoiesis resemble changes usually found in MDS or MDS/MPN and they may indicate a functional derangement possibly related to hyperinflammation/cytokine release syndrome [22]. “Aberrant” cells such as Pelger‐Huët neutrophils or monocytes are very likely to be non‐/dysfunctional leading to a high susceptibility to secondary bacterial or fungal infections representing an additional serious threat to the life of patients, especially for the critically ill [23, 24].

In addition to the immune dysregulation, a further characteristic clinical feature of COVID‐19 is the high incidence of thromboembolism [25]. A perturbation of the plasmatic coagulation as well as endothelial dysfunction possibly caused by inflammation have been reported as causative events so far [26, 27]. The high incidence of giant platelets (independent of absolute platelet counts) found in this study might also contribute to hypercoagulability in COVID‐19. Giant platelets are also a common morphologic feature of MPNs, another group of diseases associated with a high risk for thromboembolism.

Our flow cytometry analyses showed distinct immunophenotypic alterations of T‐lymphocyte subpopulations in the COVID‐19 group consistent with recent reports [14, 28, 29]. Moreover, we found significant higher antigen expression for CD3, CD5, and CD8 on the CD8^+^ T cell subpopulation in the COVID‐19 group as compared to healthy controls that may reflect a hyperactivation of CD8^+^ T cells in severe SARS‐CoV‐2 infection. Significantly elevated CD8 expression on T cells has also been observed in a recently published COVID‐19 cohort and others described that T‐lymphocytes can also be directly infected by SARS‐CoV‐2 [30, 31].

The occurrence of multi‐lineage morphologic changes in the peripheral blood gives rise to the question how hematopoiesis, in particular hematopoietic progenitor cells, could be involved in COVID‐19 pathogenesis: as a direct target of SARS‐CoV‐2 via infection and/or indirectly via pro‐inflammatory cytokine burst. The fact that only mild morphologic changes in the granulopoiesis like the left shift improved along with decreasing systemic inflammation in the “after negative SARS‐CoV‐2 test” cohort points out that there is a prolonged alteration of the hematopoiesis beyond short‐term inflammation. Thus, systematic long‐term follow up will be very important to analyze the recovery of the hematopoietic system. Particularly for patients starting/resuming cytostatic or immunosuppressive therapy this should be a prerequisite. This will also allow investigating if COVID‐19 patients have a higher susceptibility for secondary hematologic or other immune diseases.

The observed complexity and the extent of multi‐lineage morphologic changes are not found in any other virus infection, thus here constituting a hematologic COVID‐19 pattern that seems to be clinically suitable for a systematic scoring system. Since peripheral blood smears are easily obtained, rapidly performed, and cost‐effective, this approach would be particularly helpful when SARS‐CoV‐2 RNA testing is not available (for instance in developing countries). Complementary morphologic analysis might also corroborate diagnosis in cases with typical pulmonary infiltrates by computed tomography (CT) scan and negative SARS‐CoV‐2 PCR test. Moreover, the peripheral blood smears could also easily be used for follow‐up analysis and long‐term evaluations investigating the impact on the hematopoietic system. Thus, one clinical implication of our findings could be to further investigate this pattern in a larger cohort of patients at different time points in order to develop a morphologic scoring system that assists the diagnosis and follow‐up of SARS‐CoV‐2 infection. For validation purposes, for example, a blinded study evaluation of blood differentials in a general population presenting in the emergency room with respiratory symptoms could be one approach. Since little or almost nothing is known about hematologic changes in patients with asymptomatic or mildly symptomatic SARS‐CoV‐2 infection, this cohort should also be investigated. To discriminate between virus specific changes and alterations due to systemic inflammation or secondary infection a comparison our observations in COVID‐19 patients to patients in ICU care due to a different severe respiratory infection like influenza would be very helpful as well as longitudinal analysis on the temporal occurrence in the course of disease.

To the best of our knowledge, this is the first prospective study reporting a hematologic COVID‐19 pattern consisting of aberrations in all blood cell lineages. Primarily, the observed changes further illustrate the complex immune dysregulation induced by COVID‐19 and the involvement of the hematopoietic system in the pathogenesis of the disease. Second, this pattern might be further investigated and validated by a blinded, unbiased approach in a larger cohort giving rise to the development of a morphologic COVID‐19 scoring system.

## AUTHOR CONTRIBUTIONS

FL, AR, TP, and DH conceived the idea, designed the study, analyzed and supervised the study, and take responsibility for the integrity of data and the accuracy of data analysis. FL, HP, DL, ML, BS, FH, FH, BS, and DH collected and analyzed the epidemiological and clinical data. FL, JK, MG, DW, AR, and DH performed the light microscopy of smears. CB and ME performed the electron microscopy. EO and RB performed flow cytometry. FL, EO, WH, HP, TP, AR, and DH drafted the manuscript. All authors critically revised the final manuscript and are accountable for all aspects of the work, ensuring that questions related to the accuracy or integrity of any part of the work are appropriately investigated and resolved.

## CONFLICT OF INTEREST

The authors declare no competing interests and no financial support for this study.

## Supporting information

Supporting information
