# Supplementary material for: Coronavirus disease 2019 induces multi‐lineage, morphologic changes in peripheral blood cells
Source: EJHaem. 2020 Jun 29;1(1):376–83. doi: 10.1002/jha2.44 (PMC7361732; doi:10.1002/jha2.44)
Supplement: Supplementary file 1 — Supporting information [file JHA2-1-376-s001.docx]

**Supplementary Material**

**Table of Contents**

| **Supplementary Methods** |  | **1** |
| --- | --- | --- |
| **Supplementary Table I** |  | **2** |
| **Supplementary Table II** |  | **3** |
| **Supplementary Figure 1** |  | **4** |
| **Supplementary Figure 2** |  | **4** |
| **Supplementary Figure 3** |  | **5** |
| **Supplementary Figure 4** |  | **5** |

**Supplementary Methods**

**Immunophenotyping of circulating lymphocyte subsets**

Multiparametric immunophenotyping of lymphocytes in peripheral blood was carried out in altogether 23 probands, classified in 2 groups. In the first group 15 patients with severe COVID-19 and invasive mechanical ventilation were enrolled (COVID-19 group, male:female ratio: 2·0, age: 57·9 ± 11·8 years). The second group consisted of 8 healthy volunteers without any viral infection (Healthy control group, male:female ratio: 1·7, age: 55·6 ± 6·0 years). Percentages and absolute counts of major lymphocyte subsets, including T, B, NK, CD4+ T and CD8+ T lymphocytes, were determined by using TruCountTM tubes and the BD MultitestTM 6-color TBNK reagent platform (both BD Biosciences, Heidelberg, Germany), according to the instructions of the manufacturer. In addition, the surface expression of CD5 was investigated with saturating amounts of fluorophore-conjugated mouse IgG2a against human CD5 (Clone L17F12, BD Biosciences). The samples were analysed with a FACSCanto II flow cytometer by using the FACSCanto clinical software version 2·4 or the FACSDiva software version 2·0 (BD Biosciences). Antigen expression densities were assessed by mean fluorescence intensity (MFI) values for each marker.

**Statistical analysis**

We present continuous measurements as median or mean and categorical variables as count (%). For laboratory results, we also assessed whether the measurements were outside the reference range. Data were analysed by using GraphPad Prism version 7·05 (GraphPad Software, San Diego, CA, USA). t-test was used to assess significant differences between the groups. Statistical analysis of flow cytometric data was performed using the SPSS software version 26. The equality of variances for all lymphocyte variables were checked by a Levene’s test. Differences between the groups were tested by a one-way analysis of variance (ANOVA) and checked individually post hoc using a Gabriel’s test. All data are presented as mean ± S.D. P-values of *< 0·05, ** < 0·01 and ***< 0·001 were accepted as statistically significant.

**Supplementary Table I:** List of morphologic aberrations used to evaluate COVID-19 blood smears.

| **Nuclear findings** | **Cellular/cytoplasmatic findings** |
| --- | --- |
| *Aberrant lymphocytes* | |
| - (multi-) lobulated or gyrated nuclei - irregular, blurred nuclear margin - immaturity of the nucleus including a visible nucleolus - condensed/clumped chromatin - eccentric nucleus (plasmocytoid cells) - paranuclear halo (plasmocytoid cells) - nuclear vacuolation i.e. beginning karyolysis | - intense cytoplasmatic vacuolation - irregular cell outline - presence of plasmocytoid cells - presence of plasmablasts |
| *Aberrant monocytes* | |
| - irregular nuclei with partly bizzare lobulation - delicate chromatin pattern - increased nucleus:cytoplasma ratio | - Cytoplasmic basophilia - abnormal granulation - increased vacuolation |
| *Left shift* | |
|  | - Presence of blasts, promyelocytes, myelocytes, metamyelocytes |
| *Aberrant granulocytes* | |
| - hypolobulated neutrophils (Pelger-Huët neutrophils) | - hypergranulation - hypogranulation - cytoplasmic vacuolation - Döhle bodies |
| *Aberrant erythrocytes* | |
|  | - Polychromasia - Anisocytosis - Macrocytes - Microcytes - Stomatocytes - target cells - basophilic stipling - Pappenheimer bodies - Rouleaux formation - nucleated red blood cells |
| *Aberrant thrombocytes* | |
| - 'bare' megakaryocyte nuclei | - Anisocytosis - giant (vacuolated) platelets; |
| *Atypical basophils* | |
|  | - large, dark, abnormally coarse cytoplasmic granulation |
| *Apoptotic cells* | |
| - karyolyis; karyorrhexis |  |

**Supplementary Table II:** Immunophenotypic parameters of lymphocytes in the two study groups.

|  | **Measured parameter as mean ± S.D.** | | ***P* values** |
| --- | --- | --- | --- |
|  | **(A)**  COVID-19 group  n = 15 | **(B)**  Healthy control group  n = 8 | A compared to B |
| **T lymphocytes**  (% of leucocytes) | 9 5 ± 6·02 | 18 1 ± 4·05 | **0**·**005** |
| **T lymphocyte count**  (x 10^6^/L) | 1162 ± 628 | 1770 ± 202 | 0·063 |
| **CD3 expression density on T cells**  (MFI in arbitrary unit) | 5554 40 ± 427 82 | 4740 75 ± 397 37 | **0**·**001** |
| **CD5 expression density on T cells**  (MFI in arbitrary unit) | 19833 87 ± 2780.95 | 18450.25 ± 2614 31 | 0·534 |
| **CD4+ T cell count**  (x 10^6^/L) | 798 ± 390 | 1201 ± 243 | 0·063 |
| **CD3 expression density on CD4+ T cells**  (MFI in arbitrary unit) | 6607 40 ± 2947 35 | 5160 13 ± 319 05 | 0·323 |
| **CD4 expression density on CD4+ T cells**  (MFI in arbitrary unit) | 17681 00 ± 1658 13 | 15385 50 ± 3135 48 | 0·108 |
| **CD5 expression density on CD4+ T cells**  (MFI in arbitrary unit) | 22175 47 ± 2668 41 | 22225 00 ± 2388 99 | 1·000 |
| **CD8+ T cell count**  (x 10^6^/L) | 353 ± 286 | 534 ± 240 | 0·416 |
| **CD3 expression density on CD8+ T cells**  (MFI in arbitrary unit) | 4557 33 ± 501 22 | 3714 13 ± 302 07 | **0**·**001** |
| **CD5 expression density on CD8+ T cells**  (MFI in arbitrary unit) | 15025 47 ± 2550 78 | 11723 63 ± 2139 05 | **0**·**006** |
| **CD8 expression density on CD8+ T cells**  (MFI in arbitrary unit) | 5610 73 ± 771 77 | 4499 88 ± 479 60 | **0**·**002** |
| **B lymphocyte count**  (x 10^6^/L) | 214 ± 167 | 235 ± 61 | 0·985 |
| **NK cell count**  (x 10^6^/L) | 146 ± 70 | 327 ± 129 | **0**·**001** |

MFI: mean fluorescence intensity


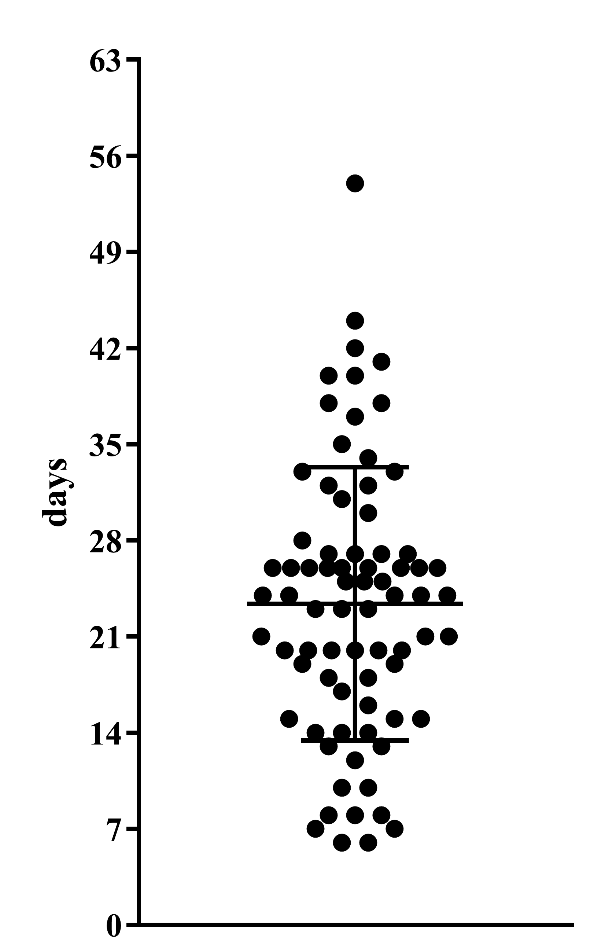


**Supplementary Figure1:** scatter dot plot of duration of COVID-19 from onset of clinical symptoms until sample collection (n=75 samples, for one patient onset of symptoms could not be determined); lines depict Mean and standard deviation;


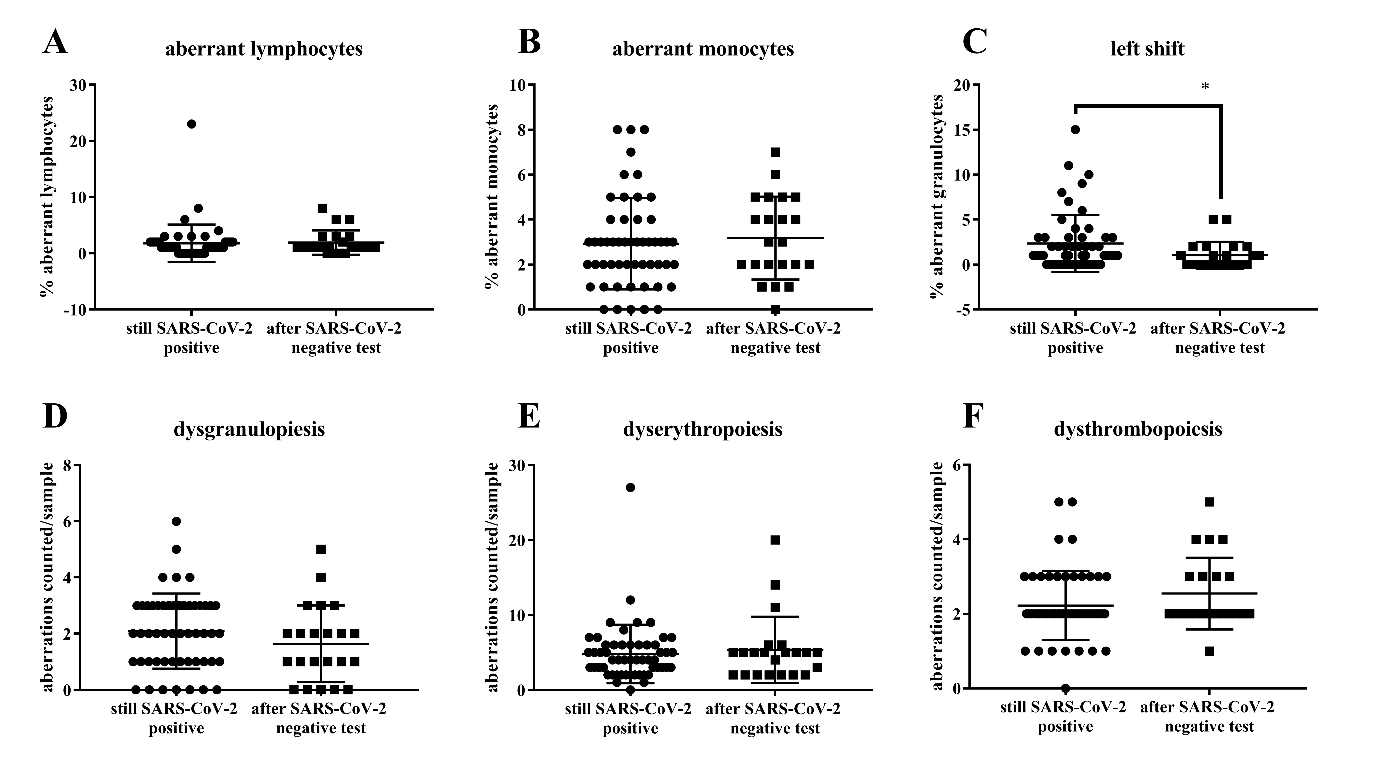


**Supplementary Figure 2:** scatter dot plots of cytomorphologic aberrations, as defined in Supplementary Table 1, in blood differentials of COVID-19 patients; (A) lymphocytes; (B) monocytes; (C) Left shift, (D) granulocytes, (E) erythropoiesis, (F) thrombocytes, horizontal lines show mean and standard deviation; * indicates p <0·05 as calculated by student’s t-test.


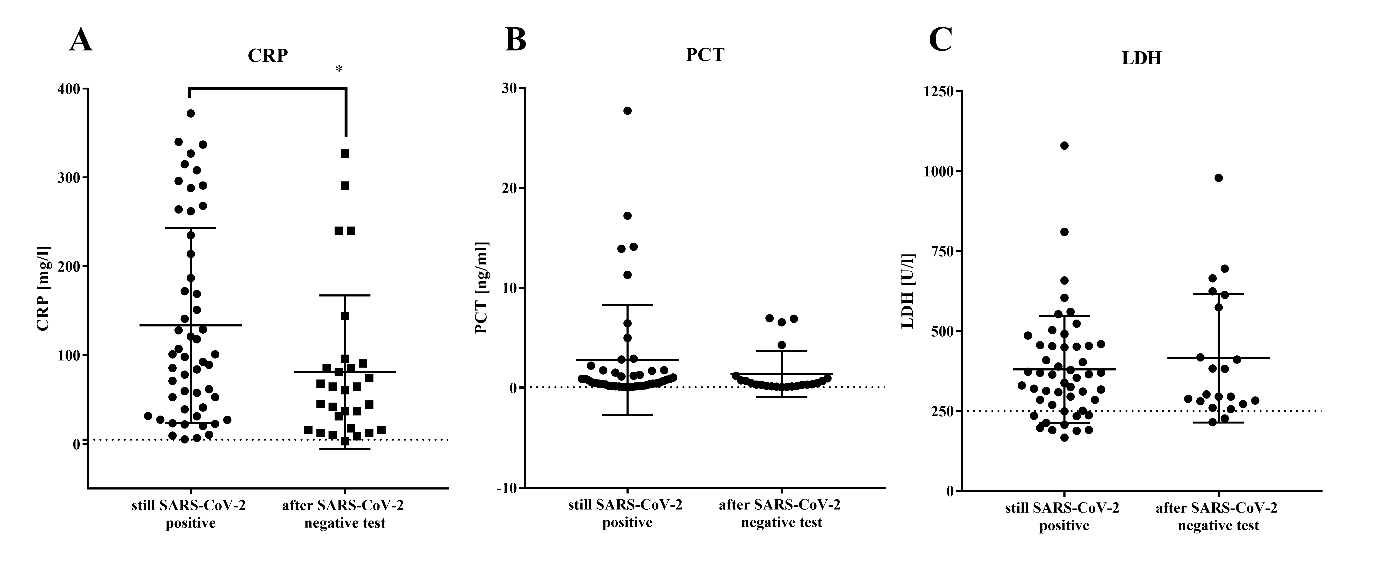


**Supplementary Fig 3:** scatter dot plots of (A) CRP, (B) PCT and (C) LDH; each dot corresponds to a blood sample subjected for morphological analysis; horizontal lines show mean and standard deviation; dotted lines depicts upper limit of normal for each parameter; * indicates p<0·05 as calculated by student’s t-test.


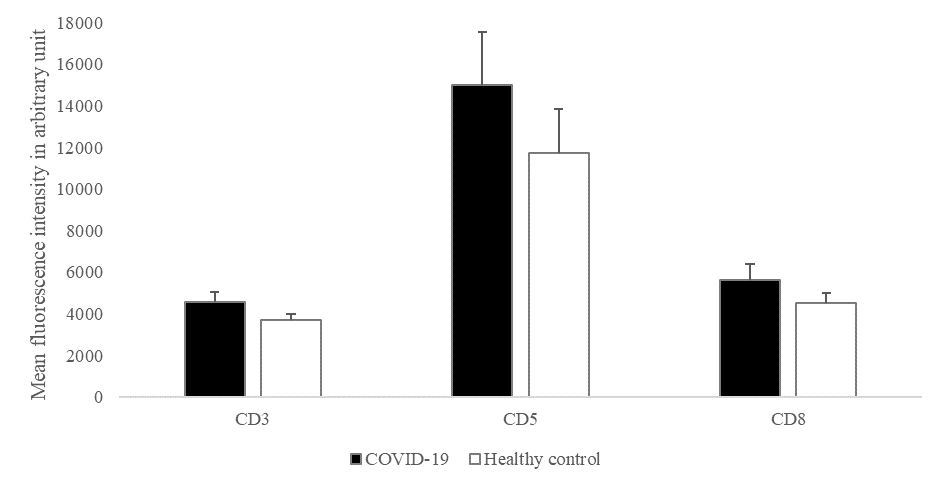


** p: 0·001

** p: 0·006

** p: 0·002

**Supplementary Figure 4:** Surface expression densities of CD3, CD5 and CD8 on circulating CD8+ cytotoxic T cells in the two study groups; Data of multiparametric flow cytometry with saturating amounts of monoclonal antibodies against human CD3, CD5 and CD8. Data are presented as mean ± S.D. Statistical significances were tested by ANOVA followed by post hoc Gabriel‘s test.
